# Virtual Standardized Patients vs Academic Training for Learning Motivational Interviewing Skills in the US Department of Veterans Affairs and the US Military: A Randomized Trial

**DOI:** 10.1001/jamanetworkopen.2020.17348

**Published:** 2020-10-15

**Authors:** Greg M. Reger, Aaron M. Norr, Albert “Skip” Rizzo, Patrick Sylvers, Jessica Peltan, Daniel Fischer, Matthew Trimmer, Shelan Porter, Pamela Gant, John S. Baer

**Affiliations:** 1 Veterans Affairs (VA) Puget Sound Healthcare System, Seattle and Tacoma, Washington; 2 Department of Psychiatry and Behavioral Sciences, University of Washington School of Medicine, Seattle; 3 Veterans Integrated Services Network 20 Northwest Network Mental Illness Research, Education, and Clinical Center, Seattle, Washington; 4 Institute for Creative Technologies, University of Southern California, Marina del Rey; 5 VA Sierra Nevada Health Care System, Reno; 6 Department of Psychology, University of Washington, Seattle

## Abstract

**Importance:**

Despite the need for effective and scalable training in motivational interviewing (MI) that includes posttraining coaching and feedback, limited evidence exists regarding the effectiveness of using virtual (computerized) standardized patients (VSPs) in such training.

**Objective:**

To evaluate the efficacy of training with a VSP on the acquisition and maintenance of MI skills compared with traditional academic study.

**Design, Setting, and Participants:**

This study was a 2-group, parallel-training randomized trial of 120 volunteer health care professionals recruited from a Department of Veterans Affairs and Department of Defense medical facility. Motivational interviewing skill was coded by external experts blinded to training group and skill assessment time points. Data were collected from October 17, 2016, to August 12, 2019.

**Interventions:**

After a computer course on MI, participants trained during two 45-minute sessions separated by 3 months. The 2 randomized training conditions included a branching storyline VSP, which provided MI skill rehearsal with immediate and summative feedback, and a control condition, which included academic study of content from the computerized MI course.

**Main Outcomes and Measures:**

Measurement of MI skill was based on recorded conversations with human standardized patients, assessed using the Motivational Interviewing Treatment Integrity 4.2.1 coding system, measured at baseline, after training, and after additional training in the randomized condition 3 months later.

**Results:**

A total of 120 volunteers (83 [69%] women), with a mean (SD) of 13.6 (10.3) years of health care experience, participated in the study; 61 were randomized to receive the intervention, and 59 were randomized to the control group. Those assigned to VSP training had significantly greater posttraining improvement in technical global scores (0.23; 95% CI, 0.03-0.44; *P* = .02), relational global scores (0.57; 95% CI, 0.33-0.81; *P* = .001), and the reflection-to-question ratio (0.23; 95% CI, 0.15-0.31; *P* = .001). Differences were maintained after the 3-month additional training session, with more improvements achieved after the 3-month training for the VSP trainees on the reflection-to- question ratio (0.15; 95% CI, 0.07-0.24; *P* = .001).

**Conclusions and Relevance:**

This randomized trial demonstrated a successful transfer of training from a VSP to human standardized patients. The VSP MI skill outcomes were better than those achieved with academic study and were maintained over time. Virtual standardized patients have the potential to facilitate dissemination of MI and may be useful for training in other evidence-based skills and treatments.

**Trial Registration:**

ClinicalTrials.gov Identifier: NCT04558060

## Key Points

**Question:**

What is the efficacy of training with a virtual standardized patient compared with traditional academic study for learning motivational interviewing skills?

**Findings:**

In this randomized trial of 120 health care professionals, training with a virtual standardized patient resulted in significantly greater improvements in 3 of 4 motivational interviewing composite skill scores, including the technical global score, the relational global score, and the reflection-to-question ratio, compared with academic study.

**Meaning:**

Virtual standardized patients offer an effective, scalable, and easy-to-disseminate training intervention.

## Teaser

This randomized trial of health care professionals evaluates the efficacy of training with a virtual standardized patient on the acquisition and maintenance of motivational interviewing skills compared with traditional academic study.

## Introduction

Standardized patients are trained actors who play patients for medical training purposes. Standardized patients are routinely used for learning and evaluation in medical school and residency but are rarely available to health care professionals (eg, physicians, nurses, and therapists) after board examination or licensure. After board examination or licensure, new evidence-based practices are adopted through continuing medical education workshops or online training. Accordingly, complex, evidence-based treatments, such as motivational interviewing (MI), are often implemented without reproducible, reliable, criterion standard skills observation and assessment.

Motivational interviewing is a directive, client-centered, evidence-based counseling approach that aims to increase patients’ motivation to make positive health changes.^1^ The style is characterized by respect for patient autonomy and an acknowledgment that ambivalence is an expected part of change. Motivational interviewing is explicitly collaborative and explores the patient’s own arguments for change as opposed to trying to convince the patient through expertise or clinical advice. Motivational interviewing has demonstrated efficacy for improving a range of health-related outcomes^2^ and is equivalent or superior to effective treatments for many health behaviors (eg, alcohol and substance use, smoking, dietary changes, gambling, unprotected sex, exercise, and treatment engagement).^3,4,5,6,7^

Improvements are needed in the training and dissemination of MI. A review^8^ found that only 2 of 11 reviewed MI training studies achieved a level of 75% of practitioners meeting a beginning proficiency standard promulgated by leading trainers.^9^ Effective training in MI typically includes expert-led workshops followed by 3 to 6 months of individual, face-to-face coaching and feedback.^10^ Miller et al^11^ compared various MI training methods and found that coaching and feedback are essential to the retention of MI skills over time, a finding consistent with meta-analytic reviews of MI training methods.^12^ However, economic analyses demonstrate the expense of such training,^13^ and adoption of such practices can be negatively affected by resource constraints or limited access to MI experts.

An alternative training approach is the use of virtual (computerized) patients that provide automated feedback and coaching after initial training. Virtual standardized patient (VSP) programs use computerized characters for standardized patient encounters. Virtual standardized patients can communicate with trainees in a highly interactive and engaging manner and can be embedded within traditional training to align with key theories of learning.^14^ Traditional training (ie, in-person workshops and online courses) includes learning concepts and demonstrations of MI skills. Typically, these skills are practiced via role-playing with peers or instructors. After training, these skills can be further rehearsed and practiced via the VSP, providing immediate coaching when errors are made and summative evaluations after simulated encounters. Virtual standardized patients have many advantages over traditional posttraining coaching and feedback, including long-term cost savings; the potential for increased availability and scalability; anytime, anywhere practice; a diverse range and complexity of standardized scenarios that can be systematically tailored to training needs; and highly replicable student experiences. Virtual standardized patients have been developed to support a wide range of medical communication training needs, including assessment of patients with posttraumatic stress disorder,^15^ medical history taking,^16^ and suicide risk assessment.^17^

Despite enthusiasm for VSPs^18^ and their incorporation into many educational health care settings,^19^ most evaluations have focused on pilot studies of feasibility or acceptability.^20,21,22,23^ Little is known about whether VSPs are more or less effective than other scalable self-directed, posttraining learning methods, such as independent study. This study compared the efficacy of a VSP program for MI skill development and retention with the efficacy of control training. In addition, given the potential role of a VSP for ongoing MI consultation and coaching, outcomes were assessed at a 3-month follow-up training encounter. We hypothesized that, compared with those assigned to training in the control condition, health care professionals trained with the VSP would demonstrate significantly greater posttraining and 3-month follow-up training in the following areas: (1) MI skills, (2) MI knowledge and reflective listening skills, and (3) self-reported MI knowledge, skills, confidence, and self-efficacy. Finally, we hypothesized that those trained with the VSP would demonstrate significantly greater training satisfaction compared with those in the control condition.

## Methods

### Study Design

This study was a single-blind, 2-group, parallel-training randomized trial that compared VSP training with control training in MI. A more extensive description of the study is available in the trial protocol in Supplement 1. The Veterans Affairs (VA) Puget Sound Healthcare System Institutional Review Board (IRB) reviewed and approved the study, and participants provided written informed consent before participation. The study followed the Consolidated Standards of Reporting Trials (CONSORT) reporting guideline. Neither the study sponsor (US Department of Defense) nor the VA institutional review board requested preregistration of this study as a clinical trial. However, at the request of the journal editors, we retrospectively registered this trial in ClinicalTrials.gov.

### Participants

**Figure 1.**
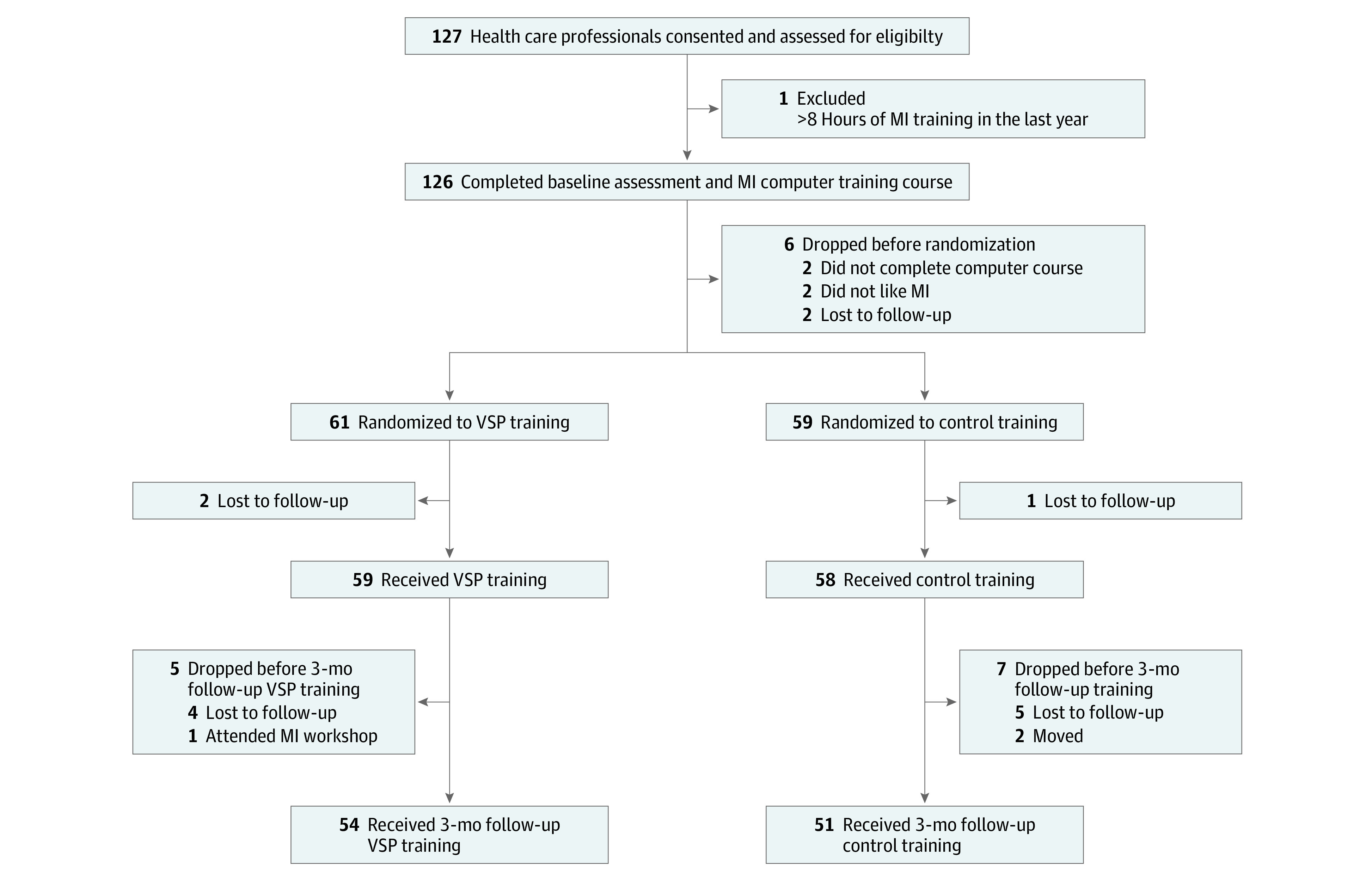
Flow of Participants Through the Study MI indicates motivational interviewing; VSP, virtual standardized patient.

Health care professionals from 2 western US medical centers participated, including a VA facility and a US Department of Defense military treatment facility. Data were collected from October 17, 2016, to August 12, 2019. The original protocol called for a sample of 114 to detect a moderate effect size (*f*^2^ = 0.11) with 80% power based on a previous trial^11^ that compared MI training methods. The sample size was increased to 126 to help account for dropouts. Six participants dropped out before randomization and were not included in the analyses. Across conditions, 95 participants (79.2%) reported some prior exposure to MI. No participant harms or unintended effects were reported. Table 1 presents demographic information, and Figure 1 presents the flow of participants throughout the study.

**Table 1.** Demographic Characteristics of the Study Participants^a^

Characteristic	Training condition	
	VSP group (n = 61)	Control group (n = 59)
Age, mean (SD), y	43.4 (10.9)	44.4 (11.7)
Female sex	39 (63.9)	44 (74.6)
Race		
American Indian or Alaskan Native	0	0
Asian	6 (9.8)	6 (10.2)
Black	4 (6.6)	5 (8.5)
Native Hawaiian or Pacific Islander	0	1 (1.7)
White	43 (70.5)	45 (76.3)
Other or mixed race	8 (13.1)	2 (3.4)
Hispanic or Latino ethnicity^b^	4 (6.6)	4 (6.8)
Highest degree obtained		
High school diploma or GED	1 (1.6)	2 (3.4)
Associate’s degree	0	2 (3.4)
Bachelor’s degree	8 (13.1)	11 (18.6)
Master’s degree	30 (49.2)	25 (42.4)
Doctoral degree	22 (36.1)	19 (32.2)
Current job or discipline		
Licensed practical nurse	0	1 (1.7)
Registered nurse or nurse practitioner	5 (8.2)	8 (13.6)
Counselor	2 (3.3)	1 (1.7)
Mental health social worker	15 (24.6)	12 (20.3)
Psychologist	13 (21.3)	13 (22.0)
Psychiatrist	3 (4.9)	1 (1.7)
Physician, nonpsychiatrist	5 (8.2)	2 (3.4)
Medical social worker	0	3 (5.1)
Other	10 (16.4)	13 (22.0)
Psychology or social work intern	8 (13.1)	5 (8.5)
Time providing patient care, mean (SD), y	12.70 (9.3)	14.56 (11.3)
Previous training with MI		
1 to 3-h presentation	34 (55.7)	29 (49.2)
4 to 8-h brief training	17 (27.9)	9 (15.3)
16- to ≥24-h workshop	8 (13.1)	3 (5.1)
Read Miller and Rollnick’s MI book^1^	6 (9.8)	11 (18.6)
Read MI journal articles	28 (45.9)	24 (40.7)
Watched MI training videos	11 (18.0)	17 (28.8)
Current use of MI in practice		
Not at all	29 (47.5)	32 (54.2)
Infrequently, with a targeted client	15 (24.6)	11 (18.6)
Occasionally, with some clients	14 (23.0)	16 (27.1)
Regularly, with many or all clients	3 (4.9)	0

Abbreviations: GED, General Educational Development; MI, motivational interviewing; VSP, virtual standardized patient.

^a^ Data are presented as number (percentage) of study participants unless otherwise indicated. The χ^2^ and independent sample *t* tests revealed no significant differences between conditions on any demographic variables.

^b^ Three health care professionals did not answer the ethnicity question.

### Inclusion and Exclusion Criteria

Inclusion required participants to be health care professionals approved to use on-duty time to participate. Exclusion criteria included the following: (1) 8 hours or more of formal training in MI in the year before baseline assessment, (2) prior formal training in MI as part of the VA’s evidence-based practice dissemination program, (3) previous experience training others in MI, (4) experience conducting prior research on MI, or (5) unavailability for the full duration of the study. An approved, preconsent screening was used to determine initial eligibility.

### Randomization and Blinding

Participants were assigned to VSP or control training (1:1 allocation) using computer-generated randomization (blocks of 6). Randomization was conducted by an investigator (P.S.) not involved in any participant contact. Training assignment was concealed from participants and study staff (in sequentially numbered envelopes) until after enrollment and all baseline measures were collected. Study investigators were blinded to collected outcome data throughout the study, and no data were analyzed until the conclusion of all data collection.

### Training Interventions

#### Computerized Motivational Interviewing Course

All participants completed the VA’s 2.5-hour online course titled Brief Motivational Interviewing for Veterans. The course includes didactic instruction and videos that illustrate foundational MI content, including the spirit of MI, MI principles, the role of change talk, MI-consistent strategies, and common MI errors.

#### VSP Training

After the VA online course, half of the participants were randomized to practice these skills and principles with a VSP via the Motivational Interviewing Novice Demonstration application. The program includes 2 virtual patient encounters with Mike. Mike is an interactive computer character designed to represent a US National Guard veteran who recently returned from deployment. In the first scenario, Mike discusses the problems he is having at home, but he is not convinced that talking to a therapist is right for him. The second scenario is a follow-up appointment with Mike, which occurs a few months after the first appointment. He brings up the problems he is still having at home, which may be partially attributable to substance use. Practitioners proceeded through both scenarios and repeated the training scenarios until 45 minutes of practice was completed.

The VSP training involves a branching storyline (eFigure in Supplement 2). At each VSP pause in the conversation, participants selected 1 of 3 computer-generated response options: (1) a correct use of MI skills and principles, (2) an incorrect use, or (3) a mixed use (ie, partly consistent and partly inconsistent with MI). Participants read aloud their selected response at each point. The VSP responded (verbally and in demeanor) according to the participant’s choices, becoming more verbally defensive and less engaged in considering change (ie, less change talk) when confronted or told what to do and, in contrast, becoming more open to change when MI-consistent responses were selected. The number of encounters completed in 45 minutes varied because the scenario duration is affected by the selected responses and speed of the participant. The program provided immediate, brief feedback when response choices were made.

The software tracked each of the practice scenarios to generate a detailed after-action review based on each health care professional’s performance. The after-action review showed performance at each decision point, facilitated review of the other response options offered, and provided corresponding feedback on why a given response was ideal, mixed, or suboptimal.

#### Control Condition Training

Participants randomized to the control training studied a detailed document that summarized key learning from the computer MI training course (eAppendix in Supplement 2). At both training sessions, participants in the control condition trained for the same amount of time as the VSP participants (ie, 45 minutes).

### Training and Assessment Procedure

All participants completed 3 study visits during approximately 3½ months. The first visit included consenting, completion of baseline self-report surveys, and a 20-minute interaction with a human standardized patient (ie, an actor) who was trained to play the part of a veteran patient. The first study visit concluded with the 2.5-hour MI computer course. All participants returned 2 weeks later for a second study visit during which they participated in their randomized training condition: VSP or control training. Participants then completed posttraining surveys and a second 20-minute standardized patient interaction to assess MI skill improvement relative to baseline. At a 3-month follow-up training visit, participants again performed their previously randomized training and then completed posttraining surveys and a third 20-minute audio-recorded MI skill assessment. To ensure full participation in the randomized training condition at each visit, the research coordinator (P.G. or S.P.) observed participants. In rare instances, participants in both training conditions expressed discomfort with a silent observer in the room. To mitigate participant stress, we honored requests for the observer to leave but emphasized the importance of training for the full time and checked on participants unannounced.

### Measures

The Motivational Interviewing Treatment Integrity 4.2.1 (MITI)^9^ was the primary outcome measure in the study. The MITI is the criterion standard for assessment of practitioner MI skill and has demonstrated good psychometric properties.^24^ It is a behavioral coding system that involves observer ratings of 4 items on a 5-point Likert-type scale (with 1 indicating low and 5 indicating high) and behavior counts. The 5-point scores for cultivating change talk and softening sustain talk are averaged for a technical global summary score, and 5-point scores for partnership and empathy are averaged for a relational global summary score. Behavior counts include simple reflections, complex reflections, and questions posed to patients. These behavior counts generate 2 summary scores: the percentage of reflections that are complex reflections and the ratio of reflections to questions. Fair and good MI skill proficiency cut points, based on expert opinion, were explored for each of the 4 MITI summary scores.^9^

Participant interactions with human standardized patients were audio recorded using a portable digital recorder, then copied to a CD-ROM and mailed to expert MITI coding consultants (Denise Ernst Consulting), who were external to the research team and not involved in any other aspects of the study. The 2 consultants were blinded to the training condition of all participants and the assessment time point (baseline, posttraining, and follow-up) at which data were collected. A total of 33 recordings (10%) were randomly selected for double coding by both experts for reliability purposes. The composite measures of relational global (intraclass correlation coefficient [ICC] = 0.71), technical global (ICC = 0.61), percentage of complex reflections (ICC = 0.57), and reflection-to-question ratio (ICC = 0.86) demonstrated moderate to good interrater reliability.^25^

Secondary outcomes (listed internal consistencies are from this study’s sample) are as follows. The Helpful Responses Questionnaire (HRQ; α = .89)^26^ is an open-response questionnaire that presents 6 paragraphs of a person describing a problem. Participants are asked to write the next thing they would say if they wanted to be helpful. Written responses are coded (blinded to treatment condition) for the complexity of reflective listening. The Motivational Interviewing Self-efficacy Scale (MIS; α = .96) was developed for this study based on self-efficacy measurement principles^27^ and included 14 self-report items on MI skills that the participant rates on a 100-point scale (0 indicating cannot do; 50, moderately certain can do; and 100, highly certain can do). The Provider Knowledge, Skills, and Confidence Survey (PKSCS; α = .84), also developed for this study, included 3 items rated from 1 (strongly agree) to 5 (strongly disagree) and assessed self-reported perceived MI working knowledge, skills, and confidence. The Provider Training Satisfaction Survey (PTSS; α = .89) was adapted from a previous study^28^ of MI training. Two academic tests of MI included the Motivational Interviewing Knowledge and Attitudes Test (MIKAT)^29^ and the Motivational Knowledge Test–Revised (MKT-R), adapted from a previous training trial.^30^

### Statistical Analysis

Statistical analyses were conducted by an analyst (A.M.N.) blinded to condition labels. Three autoregressive panel models^31^ were estimated separately to evaluate the effects of the VSP training on MI skills outcomes (MITI; hypothesis 1), MI knowledge and reflective listening skills (MKT-R, HRQ, and MIKAT; hypothesis 2), and health care professional self-reports of MI knowledge, skills, confidence, and self-efficacy (PKSCS and MIS; hypothesis 3). Three follow-up autoregressive panel models were run, excluding time 2 data, to examine change in outcome variables from time 1 to time 3. All panel models were estimated in Mplus, version 7.3^32^ using full information maximum likelihood, including all 120 randomized participants, to account for missing data according to best practices for missing data replacement in randomized longitudinal trials^33^ and examined residualized change over time by accounting for prior levels of the outcome variables of interest. All endogenous variables were allowed to correlate within each time point. MITI reflection-to-question ratio scores were log transformed before analyses.^34^ In all panel models, condition was coded as 0 for control training and 1 for VSP training.

Between-group differences at time 2 in health care professional satisfaction with the VSP training compared with control training (PTSS; hypothesis 4) were examined using independent sample *t* tests at posttreatment. All *t* tests were conducted in SPSS software (SPSS Inc), version 25,^35^ and *t* tests were 2 tailed. Significant threshold was *P* ≤ .05.

## Results

**Figure 2.**
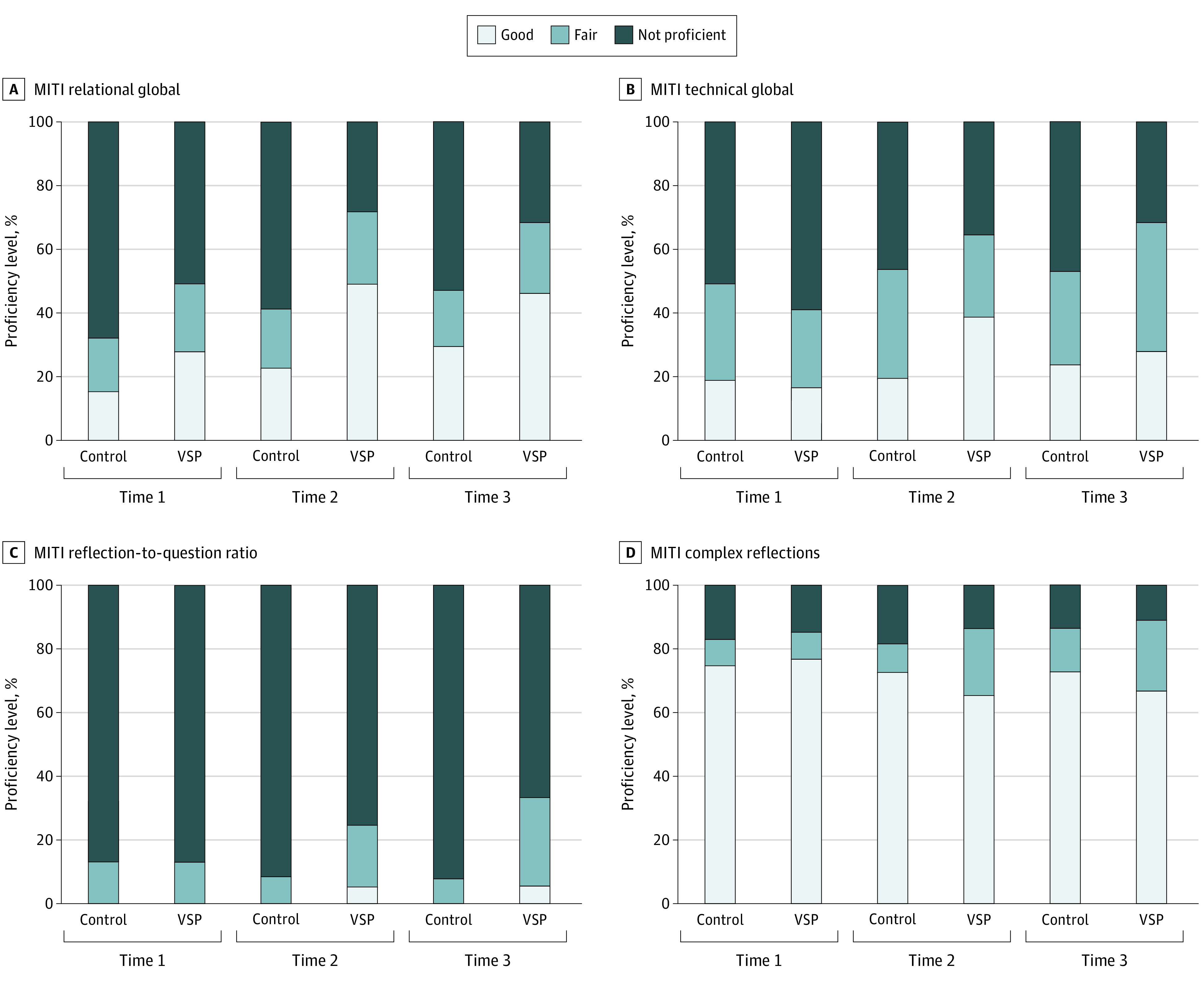
Percentages of Health Care Professionals Who Met Motivational Interviewing (MI) Proficiency Criteria at Each Time Point MITI indicates Motivational Interviewing Treatment Integrity 4.2.1; VSP, virtual standardized patient.

A total of 120 volunteers (83 [69%] women; mean [SD] age, 43.9 [11.3] years), with a mean (SD) of 13.6 (10.3) years of health care experience, participated in the study. Descriptive statistics for outcome measures are given in Table 2. Results from the 6 autoregressive panel models that evaluated the effect of study condition (VSP vs control) on change in MI skill on simulated patient (actor) interviews and all self-report measures from time 1 to time 2, time 2 to time 3, and time 1 to time 3 are given in Table 3. The percentages of participants achieving fair and good proficiency for each MI skill are shown in Figure 2. Those assigned to VSP training had significantly greater posttraining improvement in relational global scores (0.57; 95% CI, 0.33-0.81; *P* = .001), technical global scores (0.23; 95% CI, 0.03-0.44; *P* = .02), and the reflection-to-question ratio (0.23; 95% CI, 0.15-0.31; *P* = .001). Differences were maintained after the 3-month additional training session, with more improvements achieved after the 3-month training for the VSP trainees on the reflection-to-question ratio (0.15; 95% CI, 0.07-0.24; *P* = .001).

**Table 2.** Descriptive Statistics for Outcomes by Training Group and Time

Outcome	VSP group		Control group	
	No. of participants	Score, mean (SD)	No. of participants	Score, mean (SD)
MITI				
Relational global				
Baseline	61	3.02 (1.00)	59	2.79 (0.86)
Posttraining	57	3.62 (0.69)	58	2.96 (0.87)
3-Month training	54	3.50 (0.81)	51	3.13 (0.86)
Technical global				
Baseline	61	2.61 (0.69)	59	2.68 (0.68)
Posttraining	57	3.04 (0.62)	58	2.82 (0.55)
3-Month training	54	3.07 (0.61)	51	2.85 (0.72)
% Complex reflections				
Baseline	61	54.48 (13.79)	59	56.66 (16.57)
Posttraining	57	55.07 (15.23)	58	53.67 (17.59)
3-Month training	54	55.56 (13.13)	51	54.69 (18.64)
Reflection-to-question ratio				
Baseline	61	0.59 (0.34)	59	0.53 (0.34)
Posttraining	57	0.87 (0.43)	58	0.53 (0.30)
3-Month training	54	0.88 (0.45)	51	0.52 (0.28)
MKT-R				
Baseline	61	0.53 (0.12)	59	0.51 (0.12)
Posttraining	59	0.59 (0.11)	58	0.59 (0.12)
3-Month training	54	0.58 (0.13)	51	0.62 (0.11)
HRQ				
Baseline	61	11.92 (5.28)	59	10.25 (4.71)
Posttraining	59	19.00 (4.41)	58	12.43 (4.95)
3-Month training	54	19.28 (3.87)	51	12.37 (5.72)
MIKAT				
Baseline	61	23.18 (3.21)	59	22.41 (4.11)
Posttraining	59	25.56 (2.21)	58	25.53 (2.37)
3-Month training	54	25.57 (2.47)	51	25.78 (2.39)
PKSCS				
Knowledge				
Baseline	61	3.57 (1.10)	59	3.78 (0.87)
Posttraining	59	2.82 (0.80)	58	2.63 (0.93)
3-Month training	54	2.81 (1.06)	51	2.66 (1.04)
Skills				
Baseline	61	3.76 (1.12)	59	3.94 (0.86)
Posttraining	59	3.02 (0.78)	58	2.78 (0.77)
3-Month training	54	2.88 (0.78)	51	2.68 (0.75)
Confidence				
Baseline	61	3.55 (1.17)	59	3.54 (1.06)
Posttraining	59	2.97 (0.81)	58	2.70 (0.76)
3-Month training	54	2.87 (0.85)	51	2.66 (0.90)
MI self-efficacy				
Baseline	61	53.33 (19.49)	59	57.69 (22.04)
Posttraining	59	69.55 (15.34)	58	72.85 (16.38)
3-Month training	54	74.50 (14.09)	51	75.63 (14.89)

Abbreviations: HRQ, Helpful Responses Questionnaire; MI, motivational interviewing; MIKAT, Motivational Interviewing Knowledge and Attitudes Test; MITI, Motivational Interviewing Treatment Integrity; MKT-R, Motivational Knowledge Test–Revised; PKSCS, Provider Knowledge, Skills, and Confidence Survey; VSP, virtual standardized patient.

**Table 3.** Autoregressive Panel Models Examining the effect of Training Condition on Outcome Variables^a^

Outcome	b^b^ (95% CI)	β^c^
MITI		
Relational		
T1 to T2	0.57 (0.33 to 0.81)	0.36
T2 to T3	0.10 (−0.18 to 0.38)	0.06
T1 to T3	0.34 (0.06 to 0.61)	0.21
Technical		
T1 to T2	0.23 (0.03 to 0.44)	0.20
T2 to T3	0.17 (−0.06 to 0.41)	0.13
T1 to T3	0.28 (0.04 to 0.51)	0.21
Percentage of complex reflections		
T1 to T2	2.41 (−3.12 to 7.93)	0.07
T2 to T3	0.99 (−4.83 to 6.81)	0.03
T1 to T3	1.55 (−4.36 to 7.46)	0.05
Reflection-to-question ratio		
T1 to T2	0.23 (0.15 to 0.31)	0.43
T2 to T3	0.15 (0.07 to 0.24)	0.30
T1 to T3	0.23 (0.15 to 0.31)	0.45
MKT-R		
T1 to T2	−0.01 (−0.05 to 0.03)	−0.04
T2 to T3	−0.05 (−0.09 to −0.01)	−0.20
T1 to T3	−0.05 (−0.09 to −0.01)	−0.21
HRQ		
T1 to T2	5.72 (4.27 to 7.16)	0.51
T2 to T3	2.76 (0.95 to 4.57)	0.24
T1 to T3	6.23 (4.55 to 7.91)	0.54
MIKAT		
T1 to T2	−0.32 (−0.94 to 0.31)	−0.07
T2 to T3	−0.23 (−0.97 to 0.51)	−0.05
T1 to T3	−0.40 (−1.23 to 0.43)	−0.09
PKSCS		
Knowledge		
T1 to T2	0.21 (−0.09 to 0.52)	0.12
T2 to T3	0.12 (−0.27 to 0.51)	0.06
T1 to T3	0.17 (−0.22 to 0.57)	0.08
Skills		
T1 to T2	0.26 (−0.02 to 0.54)	0.17
T2 to T3	0.18 (−0.11 to 0.47)	0.12
T1 to T3	0.21 (−0.08 to 0.50)	0.14
Confidence		
T1 to T2	0.28 (−0.00 to 0.55)	0.18
T2 to T3	0.17 (−0.16 to 0.50)	0.10
T1 to T3	0.21 (−0.12 to 0.54)	0.12
MI self-efficacy		
T1 to T2	−0.67 (−4.32 to 2.99)	−0.02
T2 to T3	1.44 (−1.89 to 4.76)	0.05
T1 to T3	1.75 (−2.76 to 6.26)	0.06

Abbreviations: HRQ, Helpful Responses Questionnaire; MI, motivational interviewing; MIKAT, Motivational Interviewing Knowledge and Attitudes Test; MITI, Motivational Interviewing Treatment Integrity; MKT-R, Motivational Knowledge Test–Revised; PKSCS, Provider Knowledge, Skills, and Confidence Survey; T, time.

^a^ In all models, condition was coded as 0 for control and 1 for VSP.

^b^ Unstandardized point estimate of the effect size.

^c^ Standardized point estimate of the effect size.

Participants who received VSP training evidenced significant improvement in HRQ scores compared with the control training across all time points (time 1 to time 2: b = 5.72; 95% CI, 4.27-7.16; β = 0.51; time 2 to time 3: b = 2.76; 95% CI, 0.95-4.57; β = 0.24; and time 1 to time 3: b = 6.23; 95% CI, 4.55-7.91; β = 0.54). However, participants in the control training evidenced significantly greater improvement in MI knowledge on the MKT-R from time 1 to time 3 (b = −0.05; 95% CI, −0.09 to −0.01; β = −0.21) and time 2 to time 3 (b = −0.05; 95% CI, −0.09 to −0.01; β = −0.20), with no differences emerging from time 1 to time 2 (b = −0.01; 95% CI, −0.05 to 0.03; β = −0.04). No significant differences were found between training conditions for the MIKAT scores at any time points (b = −0.32; 95% CI, −0.94 to 0.31; β = −0.07 for time 1 to time 2; b = −0.23; 95% CI, −0.97 to 0.51; β = −0.05 for time 2 to time 3; and b = −0.40; 95% CI, −1.23 to 0.43; β = −0.09 for time 1 to time 3) (Table 3).

No significant differences were found for PKSCS self-reported perceptions of MI knowledge, skills, and confidence or for MIS scores. Finally, regarding the hypothesis that there would be significantly higher levels of satisfaction among participants who trained with the VSP compared with those in the control training group (hypothesis 4), separate independent sample *t* tests revealed significantly higher satisfaction for the training format (mean [SD] 3.33 [0.55] for the VSP group; mean [SD] 2.98 [0.75] for the control group; *t*_113_ = −2.90; *P* = .004; *d* = 0.54), training experience (mean [SD] 3.43 [0.53] for the VSP group; mean [SD] 3.19 [0.64] for the control group; *t*_113_ = −2.20; *P* = .03; *d* = 0.41), and total training satisfaction score (mean [SD], 3.38 [0.51] for the VSP group; mean [SD], 3.09 [0.67] for the control group; *t*_113_ = −2.64; *P* = .009; *d* = 0.49) among those in the VSP training group compared with those in the control training group (at time 2).

## Discussion

This randomized trial evaluated the efficacy of VSP training for MI skill development and maintenance during 3 months compared with studying the skills for the same amount of time. Findings provide support for the relative value of the VSP for training. Greater MI fidelity was found among VSP trainees compared with controls on 3 of 4 initial posttraining MI fidelity summary scores, including MI technical and relational skills, and an improved proportion of reflections to questions. All skill improvements for those assigned to the VSP group were maintained 3 months later. After additional training, the percentage of reflections to questions continued to improve compared with those assigned to the control condition. Reflective listening skills, as measured by the HRQ, also demonstrated significantly greater improvement among health care professionals who trained with the VSP. The improvement in MI skills among VSP participants by the 3-month follow-up is noteworthy. Without human instruction or an intensive training, the mean proficiency level for those in the VSP group was fair for both global summary scores, and the VSP participants demonstrated greater proficiency in the proportion of reflections to questions compared with controls as well.^9^ The VSP did not significantly impact the proportion of complex to simple reflections or objectively tested MI knowledge. Notably, those in the control group who spent time in academic study of MI demonstrated better objectively tested knowledge of MI on 1 of 2 tests but did not demonstrate improved practice with simulated patients. By comparison, those who trained with the VSP used MI with greater fidelity while also reporting significantly higher training satisfaction.

Features of VSPs may have contributed to these findings. In addition to the interactive, engaging rehearsal of learned skills, the VSP provided timely automated feedback and correction when the participants interacted in a manner that was not consistent with MI, which may have guided their successive interactions with the VSP and improved skills as the interaction proceeded. Health care professionals who trained with the VSP were also provided summary feedback at the end of VSP encounters, providing a chance to review choice points and discover how they could have engaged the VSP in a more productive manner. This summary feedback provided information on skills to maintain and areas for improvement. Trainees could immediately apply this feedback with the next VSP training interaction, which may have consolidated learning. In light of these affordances, VSPs may be uniquely positioned to assist with the resource-intensive role of providing posttraining MI feedback and coaching required for sustainment of MI skills.^11,12^

It is interesting that no significant differences between groups were seen in self-reported measures of perceived MI knowledge, skill, confidence, and self-efficacy, particularly in light of measured differences in MI skill. Although health care professionals assigned to train with the VSP had significantly greater MI skill on most primary measures, those in the control condition perceived themselves to be equally prepared. This finding highlights the importance of effective VSP MI training procedures because health care professionals may not be able to simply rely on their own assessment of their skills.^36^

### Limitations

This study has limitations. First, the VSP was used to enhance the training of a high-quality, computerized MI course. A strength of this design was the evaluation of an easy-to-disseminate, scalable MI training model. However, research should evaluate effectiveness when VSPs are used to enhance training from face-to-face MI workshops and relative to tailored, specific posttraining consultation and feedback of an expert health care professional. Second, the VSP was evaluated for maintenance of MI skills 3 months after initial training. We do not know how effective the VSP would be for longer-term feedback. Two secondary measures, the MIS and PKSCS, were developed for this study. Although they demonstrated good internal consistency in this sample, other psychometric properties are unknown. Third, participants were from the VA and US Department of Defense, and there may be systematic differences in the characteristics of this sample (ie, type of clinical experiences) that affect generalizability of the findings.

## Conclusions

This randomized trial demonstrated a successful transfer of training with a VSP to human standardized patients. These findings suggest that primary care and mental health settings could be supported with training scenarios that expand the use of VSPs in effective ways. Future research should consider the development and evaluation of additional VSP scenarios with a more complex range of MI skills. The VSP training could be expanded to more effectively train the proportion of reflections to questions. Training with VSPs could also be developed to address other topics, such as weight management, alcohol abuse, treatment engagement, exercise, and disease management. Critical public health crises, such as suicide prevention, might be supported through the use of VSPs to train health professionals in risk assessment or safety planning. Moreover, this VSP program for MI sets the stage for the development of VSP programs to train a broad range of medical communication skills or other evidence-based psychotherapies (eg, cognitive behavioral therapy).

## Notes

Supplement 1. **Trial Protocol**

Supplement 2. **eFigure.** Virtual Standardized Patient and After Action Review **eAppendix.** Control Condition Training Review: Brief Motivational Interviewing for Veterans

Supplement 3. **Data Sharing Statement**
