# Supplementary material for: Virtual Standardized Patients vs Academic Training for Learning Motivational Interviewing Skills in the US Department of Veterans Affairs and the US Military: A Randomized Trial
Source: JAMA Netw Open. 2020 Oct 15;3(10):e2017348. doi: 10.1001/jamanetworkopen.2020.17348 (PMC7563071; doi:10.1001/jamanetworkopen.2020.17348)
Supplement: Supplement 1. — Trial Protocol [file jamanetwopen-e2017348-s001.pdf]

Protocol Title: Randomized, Controlled Evaluation of a Virtual Human Patient for Provider Training of Motivational Interviewing

1.0 Introduction

The proposed study aims to evaluate the use of a virtual human simulation to reduce the use of initial, post-training patients for skill improvement during the acquisition and implementation of evidence-based psychotherapy with Department of Veterans Affairs and US Army providers. Current best practices in the training of psychotherapeutic techniques involve in-person provider workshops. After training, providers utilize learned skills in clinical practice, ideally with feedback based on coaching or supervision. Inevitably, errors are made with initial patients and Service Members and Veterans do not receive highest-fidelity therapy from newly trained providers who are, in fairness, building on their new learning through clinical experience. Opportunities to practice the application of skills prior to clinical use may help improve the quality of care. However, standardized patients (actors) are an expensive training solution that are not feasible for the training of thousands of providers in the Department of Defense (DoD) and Department of Veterans Affairs (VA). This study will evaluate an established virtual human training simulation, which was designed to support providers' acquisition of motivational interviewing skills. Following training in motivational interviewing, providers will be randomized to train with a virtual human patient or review and study the online course content. We will test the primary hypothesis that providers who practice their training with the simulation will demonstrate increased skill with their first standardized patient when evaluated by blind raters.

2.0 Objectives

Specific Aims:

To conduct a randomized controlled trial of MI training with virtual human simulation vs. a review of MI course content to evaluate effectiveness using "gold standard" training effectiveness outcomes.

Aim 1: Evaluate the pre- and post-training motivational interviewing skill of providers randomized to control and virtual human training, based on blind

expert ratings of audio recorded performance with a standardized patient (actor) interview.

Hypothesis 1a: We predict that providers trained with the virtual human simulation will demonstrate significantly greater motivational interviewing skill on simulated patient (actor) interviews relative to those randomized to a review of MI course content.

Aim 2: Evaluate the pre- and post-training motivational interviewing knowledge and reflective listening skills of providers randomized to control and virtual human training.

Hypothesis 2a: We predict that providers trained with the virtual human simulation will demonstrate significantly greater motivational interviewing knowledge and reflective listening than providers randomized to a review of their MI course learning.

Aim 3: Evaluate the pre- and post-training provider self-reports of motivational interviewing knowledge, skills, confidence, and self-efficacy.

Hypothesis 3a: We predict that providers trained with the virtual human simulation will self-report significantly greater motivational interviewing knowledge, skills, confidence, and self-efficacy with the intervention relative to those who review their MI course learning.

Aim4: Evaluate provider satisfaction with the virtual human and traditional training.

Hypothesis 4a: We predict significantly higher levels of satisfaction among providers who trained with the virtual human patient than those assigned to a review of MI course learning.

Aim 5: Evaluate the impact of virtual human training to support post-training feedback and coaching.

Hypothesis 5a: We predict that providers who receive 3-month follow-up virtual human training and coaching will demonstrate significantly greater motivational interviewing skill during simulated patient (actor) interviews relative to those reviewing MI course content.

Aim 6: A secondary aim is to evaluate the training effects of a VA on-line course on motivational interviewing.

Hypothesis 6a: We predict that providers who complete an on-line introductory course in motivational interviewing will demonstrate significantly greater motivational interviewing skill during simulated patient (actor) interviews, relative to their pre-training baseline.

3.0 Study Procedures

5.1 Study Design

Providers will participate in 3 study sessions over approximately three and a half months, although flexibility will be allowed to support provider schedules. The first visit will include consenting, completion of self-report surveys, a recorded interaction with a simulated patient (actor) to establish baseline assessment of MI skills, and completion of the on-line MI course. All participants will then return two weeks later for a second visit where they will be randomized to one of two training conditions: training with the computerized virtual human patient (i.e., MIND), or equal time reviewing content learned from the VA MI course. After completing the randomized training condition (training with MIND or review/study of TMS learning) providers will complete post-training surveys and MI assessment with a simulated patient (actor). A private space will be used for study activities, to include any data collection activities or use of the MIND software.

Pre-Consent Screening Process: To avoid unnecessary utilization of ineligible providers' time, providers who provide verbal interest in participation will be asked if they have previously completed 8-hours or more of training in Motivational Interviewing in the last year, have completed the VA Evidence Based Practice roll-out of MI, or have ever served as a MI trainer or researcher. If a provider self-reports that they have, they will be informed that they are not eligible and will be excluded. Providers will consent to study procedures following recruitment.

This study will consist of a randomized, controlled training trial in which up to 200 providers are trained in MI as part of this study. Although post-training practice with MIND is designed to replace typical "practice" on real-world first patients, a research design was needed that controlled for the additional training time and attention providers in the MIND training group would receive.

Accordingly, after the on-line computer MI training that all participants will receive, providers in this study will be randomly assigned to one of two groups: 1) virtual human patient training and feedback (MIND), or 2) a control condition - review of learning from the traditional on-line training.

Qualifying participants will then be randomized to a training condition, based on blocked, computer-generated randomization. Participants will be assessed at baseline, post-training (i.e. – after the on-line course and MIND or after the on-line course and review of on-line course content), and again at a 3-month follow-up (following another round of MIND training or review of on-line course content). The Motivational Interviewing Treatment Integrity (MITI; Moyers, Martin, Manuel, Miller, & Ernst, 2010)

will serve as the primary outcome measure in the study. Each interaction with the patient actor will be audio recorded using a portable digital recorder or straight to the study's R drive using a VA-approved microphone and Audacity software. The audio file will be transferred to the PI's secure research drive for storage, then copied to a CD or DVD and mailed to Dr. Denise Ernst Training and Consultation.

Additional measures to assess provider MI-related knowledge, and self-reported MI knowledge, skills, and self-efficacy will also be utilized. In addition, provider satisfaction with their training experience will be explored.

**Visit Number**	**Visit Components**
1.	Consent, Surveys, skill assessment with patient actor, TMS Course Completion
2.	Randomization, completion of randomized condition (MIND or TMS Course Content Review), and post-training surveys, skill assessment with patient actor
3.	3-month follow-up: completion of previously randomized condition (MIND or TMS Course Content Review), surveys, skill assessment with patient actor

Motivational Interviewing Training Protocol

After consenting, all participants will complete the VA's online course titled "Brief Motivational Interviewing for Veterans". This 2.5-hour course is hosted on the Department of Veterans Affairs Talent Management System (TMS) and is approved for ACCME continuing education (CE) for physicians, ACCME – NP for non-physicians, APA CEs for psychologists and ASWB CEs for social workers. It was developed by a nationally recognized group of subject matter experts in MI training, including one of the proposed study's co-investigators (Baer). Dr. Baer is a recognized researcher of MI training effectiveness (Baer et al, 2004; Baer et al., 2009) and he served on the faculty and planning committee for the VA course that will be used in this study.

According to the VA's on-line MI course description, the "purpose of this web course is to address the need for VHA clinicians to have the basic information on the techniques of Motivational Interviewing and to apply Motivational Interviewing to their clinical practice." The on-line course proceeds through content typical to most quality workshops on Motivational Interviewing.

We considered a research design that utilized face-to-face workshop training with a control condition that included expert follow-up consultation and feedback. Face-to-face workshop training is considered the current best practice for training providers. However, given the limited opportunities to acquire such training in the DoD (and many parts of the VA), the on-line course presents a feasible, well-established, scalable, self-training solution that represents the real-world training conditions for many providers of our Service Members and Veterans. In addition, on-line training provides a more standardized training experience for all research participants. Furthermore, the purpose of this study is to evaluate the potential for the simulation to reduce the use of real world patients in the training process of mental health providers. This need remains the same regardless of whether provider education is conducted by in person workshops or via online training.

MIND Training Protocol

The training protocol for successful implementation of MIND will follow Merrill's evidence-based adult learning principles (Merrill, 2002). After participants have learned the concepts and have seen demonstrations of MI skills and principles through the VA on-line course, participants randomized to MIND will practice the skills and principles, and receive feedback on their performance using MIND software on a laptop computer. Participants will engage in a face-to-face interaction with a virtual client using MI skills and principles as they proceed through a branching dialog conversation. Participants choose from options that reflect: 1) a correct use of MI skills/principles; 2) an incorrect use, or; 3) a mixed use. The virtual client responds according to the participant choices.

When MIND is launched, providers initially participate in a brief refresher of the key MI principles and skills summarized in the RULE and OARS acronyms, common to MI. They will then complete two virtual patient interactions with a virtual human named Mike. Mike is a National Guard veteran who recently returned from deployment. In the first scenario, Mike has come to discuss the problems he's having at home but he is not convinced that talking to a therapist is right for him. The second scenario is a follow-up appointment with Mike, which is imagined to occur a couple of months after the first. He brings up the problems he's still having at home, which may be the result of substance abuse.

During both scenarios, the encounter proceeds with a branching storyline as providers respond to a selection of multiple-choice clinical responses. The virtual human patient speaks audibly to the provider and his tone, demeanor and nonverbal behavior respond to how well, or poorly, the interaction is going based on the provider's utilization of MI skills.

The MIND software tracks the practice scenarios to generate a summarized and detailed AAR based on each provider's performance. At the conclusion of each practice scenario, the therapist is taken to the summarized review of their performance and will subsequently proceed to a detailed AAR that shows performance at each decision point, facilitates review of the other response options offered, and provides corresponding feedback on why a given response was ideal, mixed, or suboptimal in each instance. Data regarding provider MIND performances are stored and available for research purposes and will be utilized in the current study as noted above.

Control Training Protocol

To control for the amount of time and attention directed at MI content and principles, providers randomized to the control training will spend the same amount of time required for MIND participation reviewing written notes summarizing the content of the on-line training course.

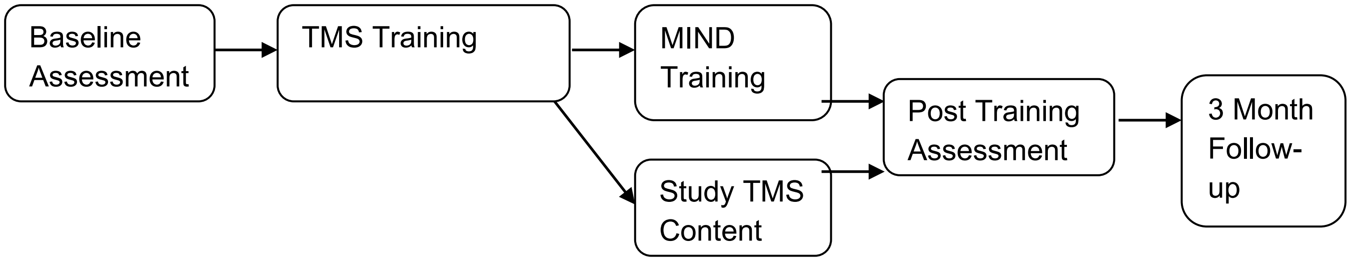

5.2 Recruitment Methods

Recruitment

Providers will primarily be recruited through direct face-to-face invitation, presentations during routine team meetings coordinated with supervisors, and email dissemination of a recruitment flyer by team leads. Interested providers during face-to-face interactions will also receive a printed study brochure with study details and contact information.

The study P.I., Dr. Greg Reger, will be blind to study subjects' identity and will not be involved in recruitment activities for all VA providers, given his leadership role within the facility. Dr. Reger will assist with recruitment of providers from the Army and forward all interested provider information from that site to the research coordinator. Co-investigators who supervise staff will not be involved in the recruitment of their direct reports.

5.3 Informed Consent Procedures

Written informed consent will be obtained prior to the baseline session of the research study. All study personnel conducting informed consents have been trained in human subjects' protections per VA R&D requirements.

5.4 Inclusion/Exclusion Criteria

All health care staff (e.g., physicians, nurses, social workers, psychologists) from supporting service lines are eligible to participate in the study.

Participants will be excluded from participation if they have completed 8 hours or more of formal training in MI in the year prior to baseline assessment. Participants will also be excluded if they have successfully completed participation in the VA Evidence Based Practice roll-out of MI. Providers will also be excluded if they have served as MI trainers or have conducted research on MI at any time. Finally, providers who do not anticipate being available for the full duration of the training study (according to their verbal self-report) will be excluded.

5.5 Study Evaluations

Measures

Primary Outcome

Motivational Interviewing Treatment Integrity 4.2.1 (MITI; Moyers, Manuel, & Ernst, 2015). The MITI is the primary outcome for this study. It is the most widely used assessment for MI skill and will be used to code segments of recorded interactions with the simulated patient actor. Global scores are computed for MI Spirit (Average of Evocation, Collaboration, Autonomy/Support), Direction, and Empathy. Coders also score MI behavior counts, which results in the Reflection to Question Ratio, Percent Open Questions, Percent Complex Reflections, and Percent MI-Adherent. Providers can be categorized based on cut points for beginning proficiency and competency. The MITI has demonstrated good reliability and validity (Moyers et al., 2005; Pierson et al., 2007). Dr. Denise Ernst is one of the developers and co-authors of the MITI and is a consultant on the proposed study. Dr. Ernst will lead the coding of the MI interactions for participants.

Secondary Outcomes

Motivational Interviewing Knowledge and Attitudes Test (MIKAT; Leffingwell, 2006). The MIKAT is a 14-item, true-false test of MI consistent statements vs. common "myths".

The measure concludes with a list of MI consistent, inconsistent, or neutral statements and the provider is asked to identify the correct MI principles. The measure has demonstrated good validity in previous MI training studies (Leffingwell, 2006).

Motivational Knowledge Test – Revised (MKT-R). The MKT-R is an 18-item multiple choice test of knowledge on motivational interviewing. Each item presents 5 options, testing participant's knowledge of core constructs and skills.

Helpful Responses Questionnaire (HRQ; Miller, Hedrick, & Orlofsky, 1991). The HRQ is an open response questionnaire that presents six paragraphs representing discrete things people with a problem might say. Participants are asked to write the next thing they would say if they wanted to be helpful. Written responses are coded for the complexity of reflective listening. The HRQ has demonstrated good reliability and validity (Miller, Hedrick, & Orlofsky, 1991) and is a frequently used measure of training outcomes in studies of MI (Baer et al., 2004; Miller et al., 2004; Shafer, Rhode, Chong, 2004).

Motivational Interviewing Self-Efficacy (MIS). Self-efficacy measures a set of self-beliefs linked to distinct realms of functioning (Bandura, 2006). Accordingly, previous studies examining health care provider self-efficacy for a particular clinical skill set utilized surveys designed for the purposes of each study. No appropriate, existing, validated self-efficacy measure for providers utilizing MI has been identified. Accordingly, this study carefully reviewed previously used self-efficacy items to adapt and develop an assessment of provider MI self-efficacy and design a survey to appropriately sample this content domain.

Provider Knowledge, Skills, and Confidence Survey (PKSCS). A survey designed for the purposes of this study was designed to assess participants' self-reported MI knowledge, clinical experience/skills, and confidence in the delivery of MI.

Provider Training Satisfaction Survey (PTSS). A survey designed for the purposes of this study will assess participants' satisfaction with the training provided and the degree to which the training enhanced their abilities and preparedness to use MI with a patient. This will include an opportunity to evaluate the training and provide feedback.

Wong and Law Emotional Intelligence Scale (WLEIS). The WLEIS is a 16-item likert-style survey to assess people's ability to appraise, express, and recognize emotions in self and others and the ability to regulate and constructively make use of those emotions. Responses on the WLEIS range from 1 (strongly disagree) to 7 (strongly agree). This measure has previously demonstrated adequate psychometric properties (Law, Wong, and & Song, 2004).

Demographics and Provider Clinical Experience Survey. Provider demographics and self-reported history of clinical training and experience history will be collected to support exclusion criteria and to consider appropriate covariates.

MIND Data. Provider responses to the clinical choice points encountered during training with MIND are collected by the software and will be used in this study.

	**Study Session**		
**Study Measure**	**Baseline**	**Post-Training**	**3 Month Follow-up**
Demographic Questionnaire	X		
Provider Clinical Experience	X		
WLEIS	X		
MITI	X	X	X
MIKAT	X	X	X
MKT-R	X	X	X
HRQ	X	X	X
MIS	X	X	X
PKSCS	X	X	X
PTSS		X	X
MIND Data		X	X

5.6 Power Calculation

For our power analysis, we examined effect sizes from research studies comparing forms of training in motivational interviewing that used our primary outcome measure (MITI). Well-designed prior research has compared self-study, workshops, workshops tailored to trainees' work context, and workshops with post-training feedback and coaching (Baer et al., 2004; Miller et al., 2004; Moyers et al., 2008; Smith et al., 2012). Our study compares an MI on-line course to the MI on-line course with the addition of MILES training, controlling for time and attention to reviewing learning. A conservative comparison would be to Miller and colleague's (2004) comparison of those trained with a workshop to those who got a workshop with the addition of written feedback on MI performance. The effect size for MI Spirit on the MITI was d = .69. Given that MIND is a unique, innovative form of practice and feedback that has not been previously evaluated, we decided upon a conservative approach to ensure adequate power to detect an effect, if it is present, and estimated a moderate effect size.

Cohen's f2 is the appropriate effect size measure to use in the context of an F-test for ANOVA. By convention, f2 effect sizes of 0.02, 0.15, and 0.35 are termed small, medium, and large, respectively (Cohen, 1988). The results of a power analysis incorporating a conservative effect size of (f2) of .11 (estimated based on the literature cited above) and a Type-I error rate of 0.05 revealed that a total sample size of 114 subjects would ensure adequate power to detect a true effect with 80% accuracy (power).

Power Calculation

Treatment Groups (G) = 2 (virtual human, traditional training)

Study Visits (V) = 3 (Baseline, Post-training, 3-month follow-up)

Effect Size (ES) = .11

Power = 0.80

$\alpha = 0.05$

df (two-way interaction) = 4

Subjects/group = [L/(ES * (V-1))] + G = 114

5.7 Withdrawal of Subjects

We do not anticipate any circumstances which would lead to study staff withdrawing participants from the study. Participants can withdraw at any time during the study and there are no consequences to their withdrawal.

4.0 Reporting

Adverse or Serious Adverse Events will be reported to the IRB and the study sponsor, Army Research Materiel and Command, within the time specifications dictated by these authorities.

5.0 Privacy and Confidentiality

This study will not collect any participant health information.

Study consent forms will be kept in a locked filing cabinet in a locked research room. Study paper copies of data will also be stored in a separate locked filing cabinet. Consents and paper copies of data generated off site will be stored in separate, locked file cabinets in locked offices until transport to the main storage site via locked 'red bag'. Study participants will be assigned a random 4-digit number to prevent their data from being identifiable. Data will not be stored at the Army site since they only serve as a non-engaged data collection site. All data will be transported to the secure locations via locked Red Bag.

Digital files will be stored on the study PI's research R:\ drive, which is secured, password protected, and only accessible to study staff. This folder will be created after IRB approval of the study per research admin policies.

6.0 Communication Plan

Communication will be required between the VA Puget Sound investigators, the Institute of Creative Technologies collaborators, and the DoD funding agency. Communication will occur via email and telephone conversations. Documents sent via email will not include participant identifiers.

9.0 Information Security and Privacy

Study consent forms will be kept in a locked filing cabinet in a locked research room. Study paper copies of data will also be stored in a separate locked filing cabinet. Study participants will be assigned a random 4-digit number to prevent their data from being identifiable.

Participants randomized to the computerized patient training condition will also generate responses to the multiple-choice response options during training. These will be stored in the computer with the individual's unique code until the data are transferred to the study data base. The non-networked computer will be stored in a locked file in a locked room when not in use.

Digital files will be stored on the study's research R:\ drive, which is secured, password protected, and only accessible to study staff. This folder will be created after IRB approval of the study per research admin policies.

10.0 References

Baer, J. S., Rosengren, D. B., Dunn, C. W., Wells, E. A., Ogle, R. L., & Hartzler, B. (2004). An evaluation of workshop training in motivational interviewing for addiction and mental health clinicians. *Drug Alcohol Depend, 73*(1), 99-106.

Baer, J. S., Wells, E. A., Rosengren, D. B., Hartzler, B., Beadnell, B., & Dunn, C. (2009). Agency context and tailored training in technology transfer: a pilot evaluation of motivational interviewing training for community counselors. *J Subst Abuse Treat, 37*(2), 191-202. doi: 10.1016/j.jsat.2009.01.003

Bandura, A. (2006). Guide for constructing self-efficacy scales. In F. Pajares & T. Urdan (Eds.), *Self-efficacy beliefs of adolescents* (pp. 307-337). Charlotte, NC: Information Age.

Law, K. S., Wong, C. S., & Song, L. J. (2004). The construct and criterion validity of emotional intelligence and its potential utility for management studies. *Journal of Applied Psychology*, *89*(3), 483.

Leffingwell, T. R. (2006). Motivational interviewing knowledge and attitudes test (MIKAT for evaluation of training outcomes. *MINUET, 13*, 10-11.

Merrill, M. D. (2002). First principles of instruction. *Educational Technology Research and Development, 50*(3), 43-59.

Miller, W. R., Hedrick, K. E., & Orlofsky, D. R. (1991). The Helpful Responses Questionnaire: A procedure for measuring therapeutic empathy. *J Clin Psychol, 47*(3), 444-448.

Miller, W. R., Yahne, C. E., Moyers, T. B., Martinez, J., & Pirritano, M. (2004). A randomized trial of methods to help clinicians learn motivational interviewing. *J Consult Clin Psychol, 72*(6), 1050-1062. doi: 10.1037/0022-006x.72.6.1050

Moyers, T. B. Manuel, J. K., & Ernst, D. (2015). *Motivational Interviewing Treatment Integrity Coding Manual 4.2.1.* Unpublished manual.

Moyers, T. B., Manuel, J. K., Wilson, P. G., Hendrickson, S. M. L., Talcott, W., & Durand, P. (2008). A randomized trial investigating training in motivational interviewing for behavioral health providers. *Behavioural and Cognitive Psychotherapy, 36*(2), 149-162.

Moyers, T. B., Martin, J. K., Manuel, W. R., Miller, W. R., & Ernst, D. (2010). Motivational Interviewing Treatment Integrity 3.1.1. *Center on Alcoholism, Substance Abuse and Addictions (CASAA)*.

Moyers, T. B., Martin, T., Manuel, J. K., Hendrickson, S. M., & Miller, W. R. (2005). Assessing competence in the use of motivational interviewing. *J Subst Abuse Treat, 28*(1), 19-26. doi: 10.1016/j.jsat.2004.11.001

Pierson, H. M., Hayes, S. C., Gifford, E. V., Roget, N., Padilla, M., Bissett, R., . . . Fisher, G. (2007). An examination of the Motivational Interviewing Treatment Integrity code. *J Subst Abuse Treat, 32*(1), 11-17. doi: 10.1016/j.jsat.2006.07.001

Shafer, M. S., Rhode, R., & Chong, J. (2004). Using distance education to promote the transfer of motivational interviewing skills among behavioral health professionals. *J Subst Abuse Treat, 26*(2), 141-148.

Smith, J. L., Carpenter, K. M., Amrhein, P. C., Brooks, A. C., Levin, D., Schreiber, E. A., . . . Nunes, E. V. (2012). Training substance abuse clinicians in motivational interviewing using live supervision via teleconferencing. *J Consult Clin Psychol, 80*(3), 450-464. doi: 10.1037/a0028176.supp (Supplemental)
