# Supplementary material for: Virtual Standardized Patients vs Academic Training for Learning Motivational Interviewing Skills in the US Department of Veterans Affairs and the US Military: A Randomized Trial
Source: JAMA Netw Open. 2020 Oct 15;3(10):e2017348. doi: 10.1001/jamanetworkopen.2020.17348 (PMC7563071; doi:10.1001/jamanetworkopen.2020.17348)
Supplement: Supplement 2. — eFigure. Virtual Standardized Patient and After Action Review eAppendix. Control Condition Training Review: Brief Motivational Interviewing for Veterans [file jamanetwopen-e2017348-s002.pdf]

Supplementary Online Content

Reger GM, Norr AM, Rizzo AS, et al. Virtual standardized patients vs academic training for learning motivational interviewing skills in the US Department of Veterans Affairs and the US military: a randomized trial. Published online October 15, 2020;10(3):e2017348. *JAMA Netw Open*.
doi:10.1001/jamanetworkopen.2020.17348

eFigure. Virtual Standardized Patient and After Action Review

eAppendix. Control Condition Training Review: Brief Motivational Interviewing for Veterans

This supplementary material has been provided by the authors to give readers additional information about their work.

Virtual Standardized Patient and After Action Review

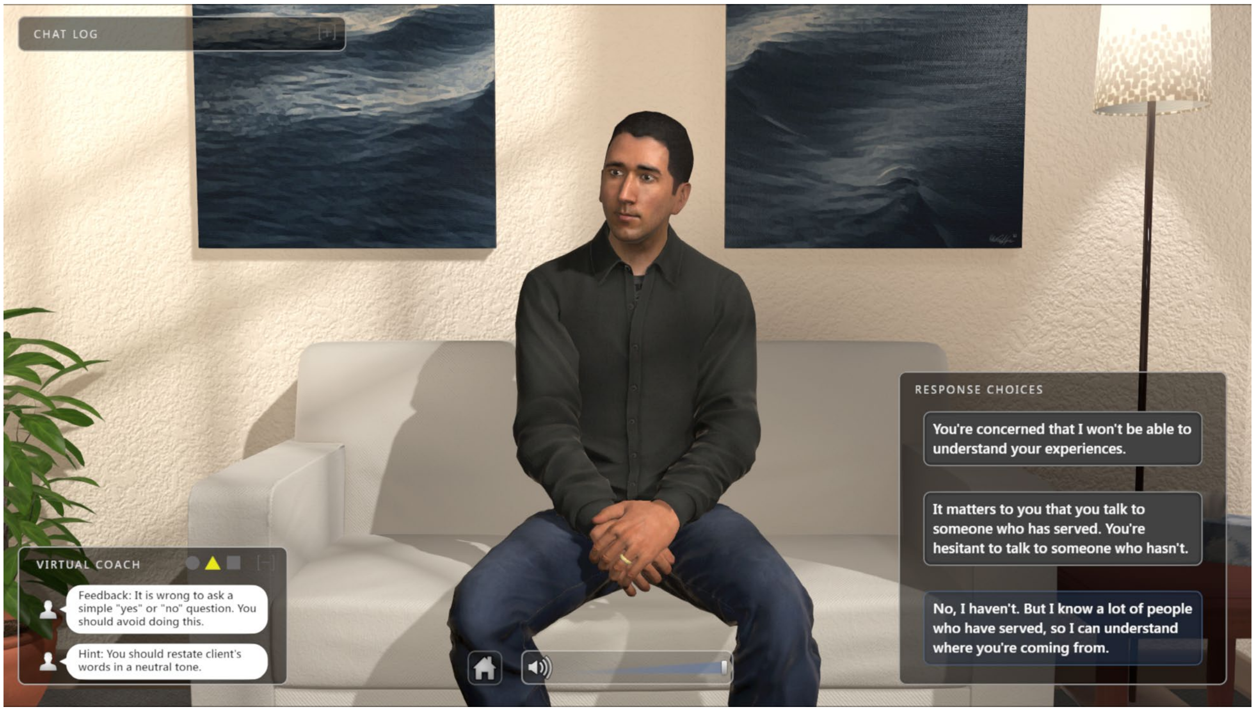

© 2020 Reger GM et al. *JAMA Network Open*

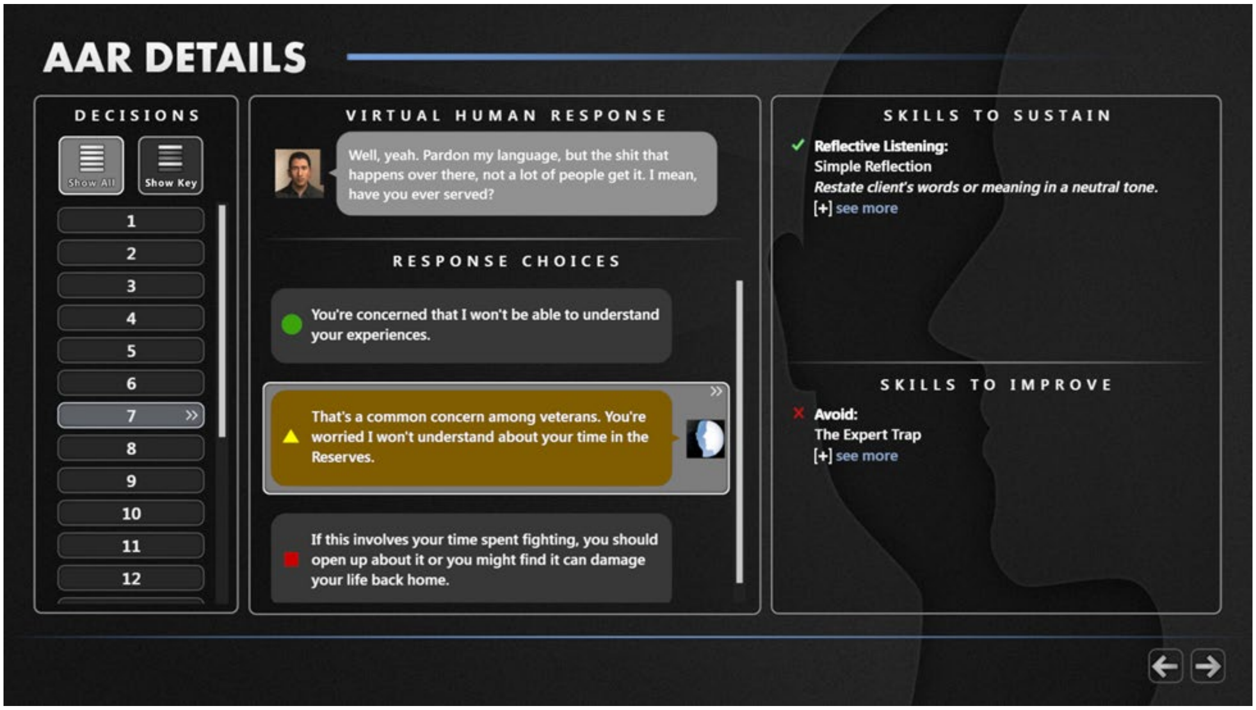

© 2020 Reger GM et al. *JAMA Network Open*

Training Review: *Brief Motivational Interviewing for Veterans*

I. MI Spirit
II. Directing, Following, Guiding Approaches
III. MI Principles (RULE)
IV. Ask Permission
V. Change Talk
VI. Starting Strategies (OARS)
VII. Rolling with Resistance
VIII. Traps to Avoid
IX. Change Planning
X. Acronym Summary

<u>Motivational Interviewing</u>: An evidence-based clinical method for eliciting and supporting a Veteran's own motivation to adopt, change, or maintain a health behavior.

- Assumes it is natural for people to have ambivalence about making changes in their substance use.

Clinicians are responsible for…

- Establishing/maintaining a collaborative, empathetic relationship
- Supporting Veteran's autonomy
- Emphasizing Veteran's responsibility for their decisions
- Understanding Veteran's perspectives without criticism/judgment
- Providing suggestions for strategies for change with Veteran's permission or when requested

Veterans are responsible for…

- Their own decisions about changing their health behaviors
- Identifying consequences of their lifestyle choices
- The desire or motivation to change
- Identifying ways to go about changing if they choose to

I. MI Spirit

- Requires adopting a "spirit" characterized by caring and collaboration, evocation of the Veteran's motivation, and support of the Veteran's autonomy and choices.
- Help the Veteran identify and articulate their own values, wishes, desires, and methods for making a positive change.

1. Support Veteran autonomy
2. Build a collaborative relationship
3. Evoke change talk

- Contrast between Motivational Interviewing and Confrontation-of-Denial Approaches:

Authoritarian Approach
Clinician argues that the Veteran must accept that he/she is an "alcoholic" or is "non-compliant" as the first step.

MI Approach
Clinician does not label the Veteran's problems. Labeling is not seen as necessary in the change process.

Educational Approach
Emphasis is on the disease process and the Veteran's lack of control or ignorance regarding the impact of the disease process.

MI Approach
Emphasis is on the Veteran's choices and capacity to gain control over the management of their condition.

© 2020 Reger GM et al. *JAMA Network Open*

Deficit Focus Approach	MI Approach
Emphasis is on the Veteran's failure to make changes or follow through with treatment.	Veteran's resistance is interpreted as a sign that the clinician may be pushing or directing Veteran to change.
**Skills Focused Approach**	**MI Approach**
The failure to make change is assumed to be a skills deficit that required remediation.	Clinician assumes that the Veteran has the capacity to make changes toward improved function and well-being.
**Authoritarian Approach**	**MI Approach**
Resistance is treated with persuasion, facts, and argumentation about the importance of change.	Resistance is met with reflective and empathetic listening or a change in strategy. Arguing with a Veteran is avoided at all costs.

II. Directing, Following, or Guiding Approach

Directing	Following	Guiding
▪ Implies uneven relationship<br>▪ Communicates that the clinician has the answer about what to do	▪ Listening predominates<br>▪ Clinician follows Veteran's lead<br>▪ Communicates: "I won't push you."	▪ A guide helps you find your way<br>▪ Communicates: "I can help you solve this for yourself."
**Examples:**	**Examples:**	**Examples:**
Take the reins	Go along with	Enlighten
Prescribe	Shadow	Motivate
Have authority	Allow	Awaken
Lead	Understand	Shepherd
Govern	Permit	Promote autonomy
Conduct	Observe	Accompany

III. MI Principles

**R**	Resist the righting reflex
**U**	Understand your Veteran's motivations
**L**	Listen to your Veteran with empathy
**E**	Empower your Veteran

© 2020 Reger GM et al. *JAMA Network Open*

1. **Resist the Righting Reflex**	▪ Guide rather than direct<br>▪ Roll with resistance
2. **Understand your Veteran's motivations**	▪ Explore ambivalence<br>▪ Evoke and affirm change talk<br>▪ Explore importance (use a 0 – 10 scale):<br><br>On a scale of 0 to 10, how IMPORTANT is it for you right now to change?<br>0___1___2___3___4___5___6___7___8___9___10<br>Not at all Important ... Extremely Important
3. **Listen to your Veteran with empathy**	▪ Use reflections<br>▪ Summarize<br>▪ Convey empathy, affirmation
4. **Empower your Veteran**	▪ Ask permission<br>▪ Elicit change strategies<br>▪ Ask—Tell—Ask<br>▪ Offer options<br>▪ Explore confidence (use a 0 – 10 scale):<br><br>On a scale of 0 to 10, how CONFIDENT are you that you could make this change?<br>0___1___2___3___4___5___6___7___8___9___10<br>Not at all Confident ... Extremely Confident<br><br>▪ Express hope for the Veteran's future

IV. Ask Permission

- Asking permission encourages the Veteran to take an active role and reduces the chances that he/she will be defensive about the conversation that follows.

V. Change Talk

**D**	Desire for change
**A**	Ability to change
**R**	Reason to change
**N**	Need for change
**C**	Commitment to change

© 2020 Reger GM et al. *JAMA Network Open*

- Instead of imposing your ideas and suggestions on your Veteran, eliciting strategies from the Veteran empowers the Veteran to use his/her own experiences and skill as the starting place for change.
- Identifying previous successes may also enhance the Veteran's confidence that they might success in the future.
- Identifying change talk:

**<u>Desire</u> for change**	"I want to do a better job of controlling my diabetes."
**<u>Ability</u> to change**	"I can do anything I set my mind to."
**<u>Reasons</u> for change**	"My doctor tells me out of control sugars hurt my whole body."
**<u>Need</u> to change**	"I am having some vision problems and my feet tingle."
**<u>Commitment</u> to change**	"I will attend the diabetes education class and learn what I need to do."

VI. Starting Strategies

**O**	Open-ended questions
**A**	Affirmations
**R**	Reflective Listening
**S**	Summaries

- The goal is to elicit and reinforce change talk

**1. Open-ended questions**	▪ Questions that encourage Veterans to talk about whatever is important to them<br>▪ Invites others to "tell their story" in their own words<br>▪ Do not *lead* in a specific direction<br><br>*Examples:*<br>▪ "How can I be of help?"<br>▪ "What are the good things about your drinking?"<br>▪ "What have you tried before?"
**2. Affirmations**	▪ Statements that recognize Veteran strengths<br>▪ Acknowledge behaviors that lead in the direction of positive change<br>▪ Strengthen clinician-Veteran relationship<br>▪ To be effective, they must be genuine and heartfelt<br><br>*Examples:*

© 2020 Reger GM et al. *JAMA Network Open*

	▪ "I really like the way you…"<br>▪ "You have a real gift for…"<br>▪ "It may not seem like much, but I think it was very impressive how you…"
3. **Reflective Listening**	▪ Listener tries to clarify and restate what the other person is saying<br>▪ Focus reflective listening on change talk and provide less attention on non-change talk<br><br>*Examples:*<br>1. **Simple reflections**<br>2. **Strategic reflections:** amplified or "agreement with a twist"<br>3. **Metaphor**<br>4. **Summary**
4. **Summaries**	▪ Effective way to communicate your interest in a Veteran<br>▪ Build rapport<br>▪ Bring attention to salient elements of the discussion<br>▪ Shift attention or direction<br>▪ *Like offering a bouquet of reflections*<br>▪ An accurate, concise summary demonstrates to a Veteran that you were listening

VII. Rolling with Resistance

- **Resistance:** A state when a clinician and Veteran are not working collaboratively together; usually, from the clinician's perspective, it feels as though the Veteran is either arguing, not listening, or being defensive.
- Usually occurs when the Veteran feels pushed toward change.
- Resistance is a call for the clinician to change, not the Veteran.

1. **Reflect it**—use **OARS** skills
2. **Shift focus**
3. **Support the Veteran's autonomy**

VIII. Traps to Avoid

1. **Expert Trap**	▪ Use the Ask—Provide—Ask approach instead: *Ask permission, provide alternatives, ask for elaboration*
2. **Labeling Trap**	▪ Clinician does not label the Veteran's problems. Labeling is not seen as necessary in the change process.

© 2020 Reger GM et al. *JAMA Network Open*

3.	**Question-answer Trap**	▪ Use open-ended questions<br>▪ Use the **3:1** ratio of **reflections to open-ended** questions to avoid this trap

IX. Change Planning

- If a Veteran remains ambivalent about change, clinicians can continue to use the MI principles and starting strategies (OARS) to help them build motivation to take action.
- **Avoid pressuring the Veteran**
- **Use the hypothetical:**
  - "If you were going to do something about your exercise, where would you start?"
- **Ask about better times and past successes:**
  - "You mentioned that you quit smoking several years back. That is really impressive. How did you do that?"

1. **Elicit specific action steps**
2. **Develop a SMART goal:**

**S**	Specific and behavioral
**M**	Measurable
**A**	Action-oriented
**R**	Realistic
**T**	Time-specific

3. **Explore confidence**
4. **Address barriers**

X. Acronym Summary:

**R**	Resist the righting reflex	**O**	Open-ended questions	**D**	Desire	**S**	Specific & behavioral
**U**	Understand Veteran's motivations	**A**	Affirmations	**A**	Ability	**M**	Measurable
**L**	Listen with empathy	**R**	Reflective listening	**R**	Reasons	**A**	Action-oriented
**E**	Empower	**S**	Summaries	**N**	Need	**R**	Realistic
				**C**	Commitment	**T**	Time-specific

© 2020 Reger GM et al. *JAMA Network Open*
