# Supplementary material for: Virtual Standardized Patients vs Academic Training for Learning Motivational Interviewing Skills in the US Department of Veterans Affairs and the US Military: A Randomized Trial
Source: JAMA Netw Open. 2020 Oct 15;3(10):e2017348. doi: 10.1001/jamanetworkopen.2020.17348 (PMC7563071; doi:10.1001/jamanetworkopen.2020.17348)
Supplement: Supplement 3. — Data Sharing Statement [file jamanetwopen-e2017348-s003.pdf]

Data Sharing Statement

Reger GM, Norr AM, Rizzo AS, et al. Virtual standardized patients vs academic training for learning motivational interviewing skills in the US Department of Veterans Affairs and the US military: a randomized trial. Published online October 15, 2020. JAMA Netw Open. doi:10.1001/jamanetworkopen.2020.17348

Data available:
No
Explanation for why data not available:
Data will be released according to VHA and DOD guidelines, upon appropriate request.
